# Nd-isotopic composition of Phanerozoic sediments in the Inner Zone of Southwest Japan Arc: implications on provenance characteristics and contribution to formation of mature island arc system

**Published:** 2004-01-01

**Authors:** Hiroo Kagami, Yoshinobu Kawano, Toshiyuki Ikawa, Naoko Nishi, Tsuyoshi Toyoshima, Takuji Hamamoto, Yasutaka Hayasaka, Yasuo Ikeda, Masaki Yuhara, Yoshiaki Tainosho

**Affiliations:** *1) Graduate School of Science and Technology, Niigata University, Ikarashi 2-no-cho, Niigata 950-2181, Japan; *2) Faculty of Culture and Education, Saga University, 1, Honjo-cho, Saga 840-8502, Japan; *3) Yachiyo Engineering Co. Ltd., Naka-Meguro 1-10-23, Tokyo 153-8639, Japan; *4) Dia Consultants Co. Ltd., Kanayama-cho 1-6-12, Nagoya, Aichi 456-0002, Japan; *5) Graduate School of Science, Hiroshima University, 1-3-2, Kagamiyama, Higashi-Hiroshima, Hiroshima 739-8526, Japan; *6) Department of Earth Science, Hokkaido University of Education at Kushiro, 1-15-55, Shiroyama, Kushiro, Hokkaido 085-8580, Japan; *7) Faculty of Science, Fukuoka University, 8-19-1, Nanakuma, Jonan-ku, Fukuoka 814-1180, Japan; *8) Department of Natural Environment, Kobe University, 1-1, Rokkodai, Nada-ku, Kobe, Hyogo 657-0011, Japan

**Keywords:** Nd model age, Paleozoic-Mesozoic sedimentary rocks, Inner Zone, SW Japan Arc, Sino-Korean Craton

## Abstract

The Nd-isotopic data on sedimentary and metamorphic rocks of SW Japan Arc allow their discrimination into five different depleted mantle (Tdm) model age clusters, 2.6-2.45 Ga, 2.3-2.05 Ga, 1.9-1.55 Ga, 1.45-1.25 Ga, 1.2-0.85 Ga. The 2.6-2.45 Ga and 1.9-1.55 Ga model ages are also coincident with U-Pb inherited zircon ages of the above two epochs as well as the major magmatic activity in the Sino-Korean Craton (SKC). The 2.3-2.05 Ga model ages can be considered as the initial formation ages for the precursors of sedimentary rocks. The Nd-isotopic data suggest that the Hida Belt was most likely formed as a part of the SKC. The mantle underlying the Ryoke Belt had continental lithospheric signature during Triassic-Jurassic period. The 1.9-1.55 Ga model ages, especially 1.8 Ga~, can be associated with the formation of this belt. The source material for the sedimentary rocks occurring in the accretionary terrane of northeastern areas in the SW Japan Arc was probably in and around the SKC of the Ryoke Belt itself. The sedimentary rocks occurring in southwestern areas of the Arc were mainly composed of materials derived from a relatively younger source (1.45-0.85 Ga).

## Introduction

**Fig. 1:**
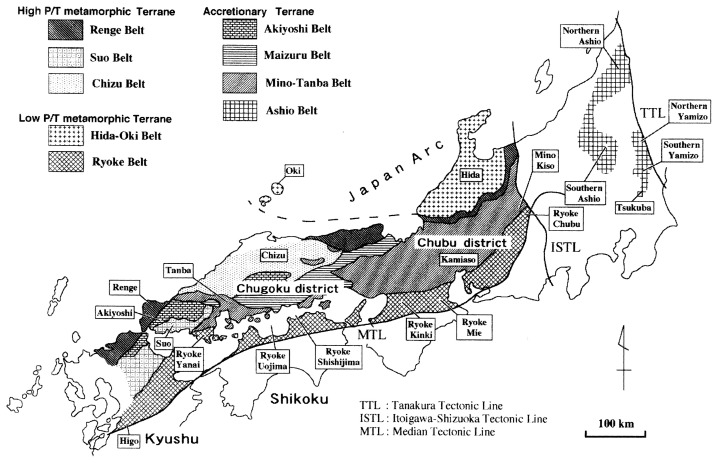
Generalized geological map1),3) of the Inner Zone (northern side of the Median Tectonic Line) of SW Japan Arc. The Ashio Belt in the NE Japan Arc is generally accepted as the northeastern extension of the Mino-Tanba Belt. The name of places enclosed by rectangles shows the sampling cites of analyzed samples.

The Japan Arc, a typical matured island arc system, had evolved through step wise accretion from the continent to the Pacific Ocean side of the eastern margin of the Eurasian Continent.1)–3) The Inner Zone of SW Japan Arc is divided into high P/T metamorphic, low P/T metamorphic and accretionary terranes. Each terrane is classified into several belts (Fig. 1). Generalized and simplified lithostratigraphic studies indicate that the Inner Zone largely consists of Paleozoic to Jurassic sedimentary and/or metamorphic rocks (except Hida Belt) accompanied with or without igneous rocks.1)–3) The Hida belt has been considered to be formed in and around the SKC.4) The Renge Belt had been accreted to the Hida Belt in Carboniferous. The Suo, Akiyoshi and Maizuru Belts had been accreted in late Permian to middle Triassic against the Hida and Renge Belts. The Chizu, Mino-Tanba and Ashio Belts and Ryoke Belt were accreted in Jurassic against the composite block which was formed by pre-Jurassic accretion. 1),3)

Initial formation ages for the precursors of sedimentary rocks and/or their originated metamorphic rocks are presumed by Nd model ages with respect to the depleted mantle (DM)5)–7) as described below. We estimate the initial formation ages of precursors for Paleozoic to Mesozoic coarse-grained sedimentary rocks occurring in the Inner Zone. This paper presents the new Nd-isotopic data and interpretation of model ages for understanding the evolution of the SW Japan Arc, whose data and interpretation can be also applied to understand similar matured arc systems.

## ^147^Sm/^144^Nd and ^143^Nd/^144^Nd ratios for calculation of Nd model ages

Chondritic uniform reservoir (CHUR) or bulk earth had been used for calculation of Nd model age at the beginning of the 1980’s, whose present ^147^Sm/^144^Nd and ^143^Nd/^144^Nd ratios are 0.1966 and 0.512638 respectively. However, recently Nd model age (Tdm) with respect to the DM have been used instead of CHUR. Several different numerical values as to the present ^147^Sm/^144^Nd and ^143^Nd/^144^Nd ratios of the DM have been used owing to the difference of its formation model.8) In this study, the Tdms were calculated using following parameters for DM (= 0 Ma) and decay constant of ^147^Sm; ^143^Nd/^144^Nd = 0.513150, ^147^Sm/^144^Nd = 0.2136, λ^147^Sm = 6.54 × 10^−12^y^−1^. Following is a brief explanation on how to calculate the Tdm.

**Fig. 2:**
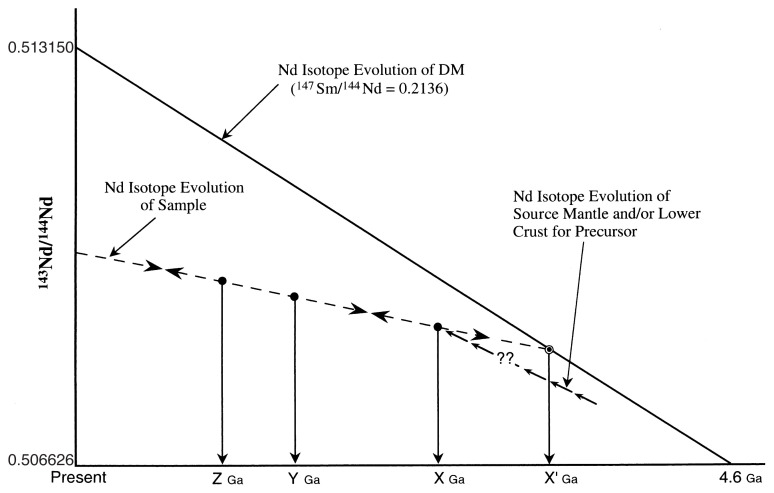
Definition of Nd model age with respect to the depleted mantle. X’ Ga; defined as Tdm, X Ga; real formative period of precursor, Y Ga; formative period (including deposition and cementation) of sedimentary rock, Z Ga; period of metamorphism. Inclination of Nd isotope evolution line corresponds to ^147^Sm/^144^Nd ratio of each sample.

Igneous rocks were formed from mantle- and/or lower crust-derived magmas (X Ga, Fig. 2). The igneous rocks formed on the earth’s surface were directly affected by weathering and erosion. Some other igneous rocks consolidated in the deep crust are exposed at earth’s surface through upheaval and then affected by weathering and erosion. Detrital grains originated from the weathered igneous rocks were mainly transported by streams and deposited at the sea, lake bottoms and so on, and hardened as sedimentary rocks through cementation and compaction (Y Ga). Some sedimentary rocks were transformed into metamorphic rocks by metamorphism (Z Ga). Passing through these geologic processes, Sm/Nd (and ^147^Sm/^144^Nd) ratios of the igneous rocks and sedimentary rocks (hereafter, including metamorphic rocks) would not be largely modified, which is a significant and fundamental assumption dealing with Nd model age. Actually, even if the Sm/Nd ratio is slightly changed, this change is impossible to be confirmed. Because the complete precursors (igneous rocks) are hardly identified on the field since they have been transformed into the equivalent sedimentary rocks. In other words, the precursors were lost from the Earth.

The formative period (Y Ga) of sedimentary rock is defined using isotope dating and fossils. And ^143^Nd/^144^Nd ratio of the sedimentary rock at the formative period can be calculated using its present ^143^Nd/^144^Nd and ^147^Sm/^144^Nd ratios and age. However, as the real formation period (i.e. X Ga) of the precursor (igneous rock) of the sedimentary rock is uncertain, the Nd isotope evolution line of the sedimentary rock, whose inclination corresponds to ^147^Sm/^144^Nd ratio, extends to the direction of that of DM. Intersection point (i.e. age) of Nd evolution lines of the depleted mantle and sedimentary rock is defined as the Tdm (X’ Ga). At this point, there is a possibility that the Tdm for the sedimentary rocks with a high inclination (i.e. high ^147^Sm/^144^Nd ratio) is largely different from real formation period (X Ga) of its precursor. In order to avoid large difference between X’ Ga and X Ga, the age is generally applied to the rocks with low ^147^Sm/^144^Nd ratio less than 0.14.6) Accordingly, this ratio is the lower the better for the calculation of the Tdm. Furthermore, the rocks with high present ^143^Nd/^144^Nd ratios have not been used because of decline of significance as to the Tdm. In this paper, the Tdms for the samples with present ^143^Nd/^144^Nd ratio less than 0.5125 and ^147^Sm/^144^Nd ratio less than 0.13 were calculated.

## Sampling and Nd-isotopic analysis

**Fig. 3:**
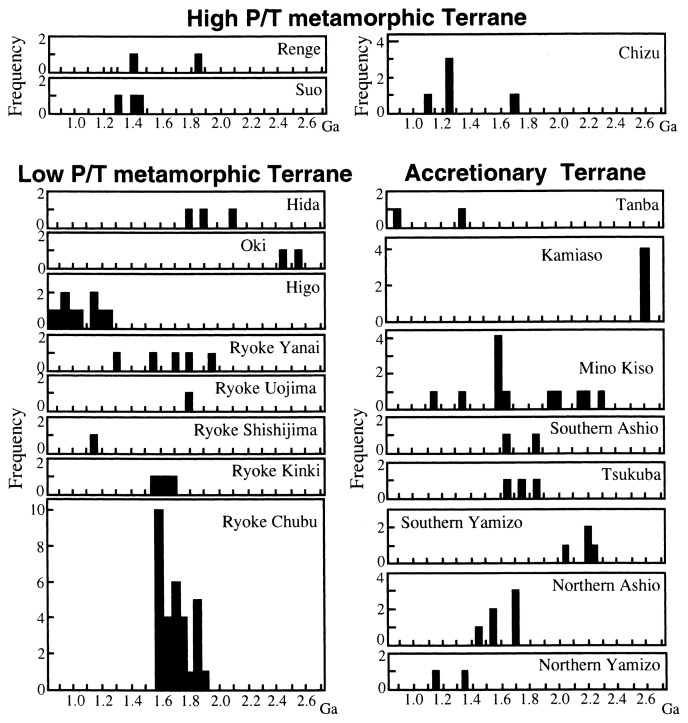
Frequencies of Tdms of Paleozoic to Mesozoic sedimentary rocks from SW Japan Arc. The Tdms are given from the data shown in Table I and the published data.

Representative argillaceous to psammitic sedimentary rocks and their originated metamorphic rocks were sampled and analyzed (Fig. 1). Their Sm- and Nd-isotopic ratios and Tdms are presented in Table I and Fig. 3. Previously published Sm- and Nd-isotopic data9)–17) have also been considered while calculating Tdm. The extraction procedures for Sm and Nd from rock powders are following Kagami *et al*. (1989).18) Isotopic analyses were performed on MAT261 and MAT262 mass spectrometers at Niigata University. The ^143^Nd/^144^Nd ratios were normalized to ^146^Nd/^144^Nd = 0.7219 and are reported relative to ^143^Nd/^144^Nd = 0.512115 for Ndi-1 (GSJ Standard) corresponding to ^143^Nd/^144^Nd = 0.511858 of LaJolla.19) Mean uncertainty of ^143^Nd/^144^Nd ratio for each sample is ca. 0.003% as 2σ-value. Sm and Nd concentrations were obtained using ^149^Sm-^150^Nd mixed spike. Analytical errors of ^147^Sm/^144^Nd ratios are 0.1% as standard deviation. The published Nd isotopic data, ^143^Nd/^144^Nd, given in Table II and Fig. 3 were recorrected using following standard samples and their values; LaJolla (0.51185819)), BCR-1 (0.51263818)), JB-1a (0.51278418)), JNdi-1 (0.51211519)).

## Isotopic data and Nd model ages

It is noteworthy that the Tdms obtained from certain belt (or district in the belt) of the terranes are not randomly scattered but cluster around restricted specific time brackets as shown in the Tdm frequency diagram (Fig. 3) and summary as shown in Table II.

### High P/T metamorphic terrane

The Tdms are divided into three age categories of ca. 1.75 Ga, 1.45-1.3 Ga and 1.25-1.1 Ga. The former two categories are coincident with U-Pb inherited zircon ages.

### Low P/T metamorphic terrane

The Tdms of Oki-Dogo metamorphic rocks are 2.55-2.45 Ga though their Sm-Nd whole rock ages27) are 1.98 Ga and 1.96 Ga. Each district of the Ryoke Belt is isolated with significantly different Tdms ages. However typical Tdms for the Ryoke Belt range between 1.9 Ga and 1.55 Ga. And the oldest Tdms (1.9 Ga) are coincident with U-Pb inherited zircon ages (1.95-1.7 Ga).

### Accretional terrane

We didn’t analyze Sm and Nd isotopic compositions of rocks from the Maizuru Belt because these are mainly volcanic rocks and volcaniclastic sediments which were formed in oceanic and island arc settings.28) The Sm-Nd and Rb-Sr whole rocks ages of Kamiaso conglomerates are 2.07 Ga13) and 2.06-1.89 Ga,29) respectively. The Tdms (2.6 Ga) of the rocks are coincident with one of the U-Pb inherited zircon ages. The Tdms in Ashio Belt cluster at 1.85-1.6 Ga except northern Ashio, northern Yamizo and southern Yamizo, which are coincident with typical Ryoke Belt and Kyogatake Complex (Kiso in the Mino Belt). The Tdms of southern Yamizo are completely coincident with those of the Miso-gawa Complex (Kiso).

## Discussions

Initial Nd isotopic ratios of some igneous rocks of the SKC with activity ages from mid-Archean (ca. 3.6 Ga) to mid-Proterozoic (ca. 1.5 Ga) are plotted along the Nd isotope evolution line of DM13),30) (refer to Fig. 2, as to this line). However, most of the late Archean (ca. 2.6 Ga) to late Proterozoic (ca. 0.8 Ga) granitoids and meta-sedimentary rocks from the SKC are plotted on the Nd evolution line linking 0.511510 (= ^143^Nd/^144^Nd(0 Ga)) with 0.509572 (2.6 Ga).13),30) This evolution line with an inclination of 0.113 (= ^147^Sm/^144^Nd) intersects that of DM at 2.6 Ga. Initial ^143^Nd/^144^Nd ratios of the granitoids (Tdm = 2.6 Ga) from Kamiaso conglomerate and metamorphic rocks (Tdm = 2.55-2.45 Ga) from Oki-Dogo Island are plotted on this line. Metamorphic rocks from Korean Peninsula are also plotted on the same line.31) These data suggest that the evolution line of DM is useful for interpreting the initial formation age of sedimentary rocks and their originated metamorphic rocks in the SKC. The paleogeographic configuration of such terranes (or belt or district) of the SW Japan Arc is not yet properly understood. As described above, the initial ^143^Nd/^144^Nd ratios of the rocks from Kamiaso conglomerates and Oki-Dogo Island are plotted on one of the Nd evolution lines of the SKC. Triassic to early Jurassic mafic volcanic rocks constituting the Ryoke Belt were formed from the continental lithospheric mantle.32) These two observations have led to conclusion that some belts of SW Japan Arc were formed under a continental regime. Furthermore, the analyzed samples in this study also show an affinity with active and passive continental margins as seen in some discrimination diagrams. 33) This implies that if Paleozoic to Mesozoic sedimentary rocks of SW Japan Arc were derived from the SKC or its surrounding areas, the Tdms should provide information on formation age of SW Japan Arc.

Some of the Tdm values obtained do not show any scatter but cluster around five different age brackets; (1) 2.6-2.45 Ga, (2) 2.3-2.05 Ga, (3) 1.9-1.55 Ga, (4)1.45-1.25 Ga, (5) 1.20-0.85 Ga. Their significance is discussed below.

1. 2.6-2.45 Ga; metamorphic rocks from the Oki-Dogo Island (Hida Belt) and granitic conglomerates from the Kamiaso district (Mino Belt) belong to this age category. Tdms of 2.6-2.45 Ga, especially the older one, are probably initial formation ages of the precursors of sedimentary rocks because they are close to 2.8-2.6 Ga of SKC.13),22),30) The Hida Belt is probably formed in and around the SKC as pointed out by Isozaki (1997)4) and others.
2. 2.3-2.05 Ga; sedimentary rocks from Kiso in the Chubu district (Mino Belt) and from the southern Yamizo and metamorphic rock from the Hida (Hida Belt) belong to this age category. Main Proterozoic igneous activities in the SKC have started at ca. 2.1Ga.34),35) The oldest inherited zircon CHIME ages that were given for the gneisses from southern Korea Peninsula are 2.15 Ga.36) Considering this, though the Tdms of 2.3 Ga might indicate the formation age of precursors of sedimentary rocks, and to confirm this matter it is necessary to obtain more detailed age data such as zircon ages.
3. 1.9-1.55 Ga; the belts or districts in the belt belonging to this age category are, Ryoke Belt (except for Shishijima, Mie), Mino Belt (Chubu), Hida Belt (some of metamorphic rocks), Ashio Belt (except for Yamizo). Two samples from the Renge and Tanba Belts fall under the same age category. As described above, U-Pb inherited zircon ages from the Ryoke metamorphic and igneous rocks are 1.95-1.8 Ga. These zircon ages are coincident with the older (1.8 Ga~) Tdms ages from the Ryoke Belt. In that respect, it is notable that the U-Pb zircon ages of 1.95-1.8 Ga are also recognized in other belts of SW Japan Arc. One of the Proterozoic igneous activities of the SKC took place around 2.0 Ga and 1.7 Ga34) and 2.0-1.6 Ga.37) The age category (1.9-1.55 Ga), especially older (1.8 Ga~) ages, probably indicates initial formation age of the precursors of the sedimentary rocks of the Ryoke Belt as well as the other belts of this age category. Triassic to early Jurassic mantle underlying the Ryoke Belt had continental lithospheric signatures as described above. The fundamental formation age of this belt is probably close to 1.9-1.55 Ga (or 1.8 Ga~) and it was formed in and around the SKC. The sources of sedimentary rocks occurring in the accretionary terrane (Mino and Ashio Belts, excluding Yamizo) of northeastern areas in the SW Japan Arc are probably in and around the SKC or Ryoke Belt itself. The Hida metamorphic rocks belong to age categories (2) and (3) above, however, some rocks from this belt plot close to the 0.5 Ga line as shown in Fig. 3. These data suggest that some metamorphic rocks were formed at relatively young age. Accordingly, the Hida metamorphic rocks have probably been formed from several rocks (protoliths) with various ages ranging from Proterozoic to early Paleozoic.
4. 1.45-1.25 Ga; the terranes belonging to this age category are mainly in the Renge, Suo and Chizu Belts. One of the middle Proterozoic igneous activities in the SKC is ca. 1.4 Ga.34)
5. 1.20-0.85 Ga; this age category is mainly defined by the rocks from Higo district that is generally accepted as the western extension of the Ryoke Belt (Fig. 1). However, the Tdms of Higo are quite different from those of the typical Ryoke Belt, which implies that the former is not a part of the latter as pointed out by Osanai *et al*. (1996)38) and others. According to CHIME age using zircon,39) their inherited Proterozoic ages are scattered between 1.9 Ga and 0.8 Ga and most of them are concentrated between 1.4 Ga and 0.8 Ga. The rocks from the Chizu Belt belong to both age categories (4) and (5).

**Fig. 4:**
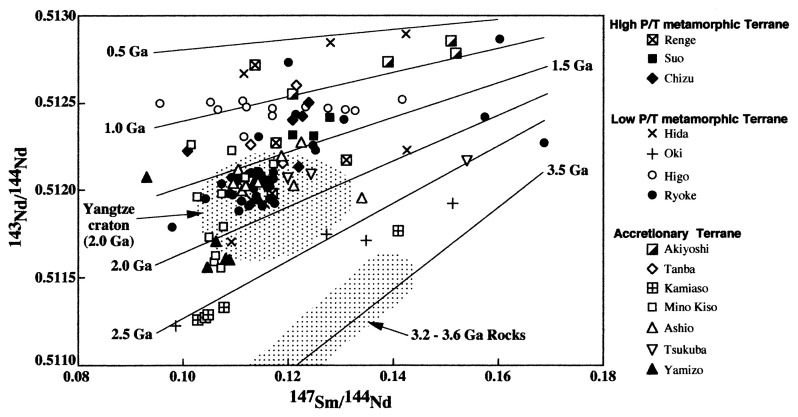
^143^Nd/^144^Nd vs. ^147^Sm/^144^Nd ratios relationship for Paleozoic to Mesozoic sedimentary rocks from SW Japan Arc. Used Sm and Nd isotopic data are given in Table I, published data (see Table II). Fields of Yangtze Craton and 3.6-3.2 Ga igneous rocks were cited from Chen and Yang (2000).7) Straight lines indicating each age are explained by following example. That is, ^147^Sm/^144^Nd and ^143^Nd/^144^Nd present ratios of the rocks formed from DM at 2.0 Ga ago are plotted on the line indicating 2.0 Ga.

Some sedimentary rocks collected from the districts belonging to (4) and (5) contain detrital zircons with old ages (ca. 1.9 Ga) as described above. If all sedimentary rocks consist of mixtures of several different components with various ages as well as detrital zircons, the obtained Tdms are of little value. However, one of the middle to late Proterozoic igneous activities in the SKC took place at ca. 1.4 Ga and 1.0-0.7 Ga.34) Furthermore, based on the high of ^143^Nd/^144^Nd ratios (> 0.5122, Fig. 4) in addition to normal ^147^Sm/^144^Nd ratios (0.13-0.10) for the sedimentary rocks belonging to age categories (4) and (5), their source materials have been considered to be formed at relatively young age. Though the Tdms of Akiyoshi Belt belonging to accretionary zone couldn’t be obtained because of high ^143^Nd/^144^Nd and ^147^Sm/^144^Nd ratios, they plot between 0.5 Ga and 1.0 Ga lines (Fig. 4). This data imply that the precursors of sedimentary rocks are young and did not have a long history. Thus, even if the sedimentary rocks were formed from the materials with various ages, most contributing materials of the sedimentary rocks should have younger ages. It is needless to say that this matter should be confirmed using other techniques such as U-Pb zircon method. Considering the ages of mid- to late Proterozoic orogeny and Sm-Nd isotopic data, the ages from 1.45 Ga to 0.85 Ga (age categories (4) and (5)) defined by rocks collected from southwestern areas (Renge, Suo and Chizu Belts, Higo district in the Ryoke Belt, accretionary terrane including Akiyoshi and Tanba Belts) in the SW Japan Arc probably indicate the formation age of the precursors of the sedimentary rocks.

## Figures and Tables

**Table I:** Sm and Nd isotopic data and Tdms

Number of Samples	Name of belts & districts	Name of Rocks	^147^Sm/^144^Nd	^143^Nd/^144^Nd	Tdm(Ga)
	**1. High P/T Metamorphic Terrane**				
1	Renge Belt	schist*	0.1166	0.511980	1.83
2		schist*	0.1305	0.512173	–
3	Suo Belt	schist*	0.1248	0.512309	1.45
4		schist*	0.1212	0.512311	1.38
5		schist*	0.1282	0.512411	1.32
6	Chizu Belt	shale	0.1006	0.512220	1.25
7		sandstone	0.1218	0.512127	1.69
8		schist*	0.1230	0.512420	1.23
9		schist*	0.1209	0.512401	1.23
10		schist*	0.1235	0.512494	1.11
	**2. Low P/T Metamorphic Terrane**				
11	Ryoke Belt (*Higo district*)	gneiss*	0.1111	0.512309	1.25
12		gneiss*	0.1110	0.512514	–
13		gneiss*	0.1054	0.512504	–
14		gneiss*	0.1166	0.512466	1.07
15		gneiss*	0.1168	0.512426	1.14
16		gneiss*	0.1312	0.512463	–
17		granulite*	0.1116	0.512476	1.01
18		granulite*	0.0953	0.512495	0.84
19		granulite*	0.1312	0.512459	–
20		granulite*	0.1229	0.512476	1.13
21		granulite*	0.1278	0.512470	1.20
22		granulite*	0.1066	0.512462	0.98
23		granulite*	0.1416	0.512521	–
24		granulite*	0.1328	0.512454	–
25	(*Yanai district*)	gneiss*	0.1853	0.512621	–
26		gneiss*	0.1144	0.512309	1.29
27		gneiss*	0.1177	0.511923	1.94
28	(*Uojima*)	gneiss*	0.0977	0.511792	1.78
29	(*Shishijima*)	gneiss*	0.1199	0.512729	–
30		gneiss*	0.1215	0.512432	1.19
31	(*Kinki district*)	mudstone	0.1091	0.511977	1.71
32		mudstone	0.1250	0.512230	1.58
33		sandstone	0.1090	0.512074	1.56
34		shale	0.1109	0.511940	1.79
35	(*Mie district*)	gneiss*	0.1605	0.512866	–
36		gneiss*	0.1681	0.512272	–
37		gneiss*	0.1304	0.512405	–
38		gneiss**	0.1572	0.512416	–
	**3. Accretional Terrane**				
39	Akiyoshi Belt	mudstone	0.1391	0.512729	–
40		shale	0.1205	0.512547	–
41		shale	0.1517	0.512780	–
42		sandstone	0.1514	0.512874	–
43	Tanba Belt (*Chugoku district*)	mudstone	0.1133	0.512256	1.36
44		mudstone	0.1191	0.512149	1.61
45	Mino Belt (*Kiso*)	sandstone#1	0.1061	0.511591	2.20
46		sandstone#1	0.1064	0.511626	2.16
47		sandstone#1	0.1073	0.511563	2.30
48		slate#2	0.1100	0.512056	1.61
49		sandstone#2	0.1171	0.512148	1.58
50		sandstone#2	0.1072	0.511985	1.67
51		slate#2	0.1027	0.511972	1.63
52		slate#3	0.1079	0.511792	1.98
53		sandstone#3	0.1051	0.511736	2.00
54		slate#3	0.1119	0.512076	1.62
55		slate#4	0.1013	0.512287	1.18
56		slate#4	0.1092	0.512233	1.35
57	Ashio Belt (*southern Ashio*)	sandstone	0.1212	0.512026	1.85
58		sandstone	0.1094	0.512038	1.62
59	(*Tsukuba*)	gneiss*	0.1244	0.512085	1.81
60		gneiss*	0.1193	0.512069	1.75
61		mudstone	0.1126	0.512047	1.66
62		sandstone	0.1542	0.512163	–
63	(*southern Yamizo*)	sandstone	0.1091	0.511602	2.25
64		mudstone	0.1047	0.511556	2.22
65		sandstone	0.1077	0.511605	2.21
66		mudstone	0.1064	0.511708	2.04
67	(*northern Yamizo*)	gneiss*	0.0931	0.512076	1.36
68		mudstone	0.0581	0.511993	1.13

* argillaceous,

** psammitic,

#1 Miso-gawa Complex,

#2 Kyogatake Complex,

#3 Shima-shima-dani Complex,

#4 Sawando Complex.

Terms of ^#1^–^#4^ are given in Takeuchi *et al*.20)

**Table II:** Tdms of the Inner Zone of SW Japan Arc

Name of belts & districts	TDMs (Ga)	inherited U-Pb zircon ages (Ga)
**1. High P/T Metamorphic Terrane**		
Renge Belt	1.85, 1.415), ^Table I^	
Suo Belt	1.45-1.3	1.91,21) 1.75,22) 1.4-1.3,22) 0.9321)
Chizu Belt	1.7, 1.25-1.1	
**2. Low P/T Metamorphic Terrane**		
Hida Belt	2.55-1.8	
*Oki-Dogo Island*	2.55-2.4510)	1.9623)
*Chubu district*	2.1-1.812)	3.42,24) 2.56,24) 1.84,24) 1.1324)
Ryoke Belt	1.9-0.85	
*Higo district*	1.25-0.8514), ^Table I^	1.78*1
*Yanai-Uojima*	1.8-1.5, 1.39), ^Table I^	1.95*2, 25)
*Shishijima*	1.2	
*Kinki district*	1.8-1.55	1.9,*3 1.86,*2, 25) 1.826)
*Mie district*	–	
*Chubu district*	1.9-1.559), 17)	
**3. Accretional Terrane**		
Akiyoshi Belt	–	2.7-2.422)
Tanba-Mino Belt	2.6-0.9	
(1) Tanba Belt (*Chugoku district*)	1.6, 1.35, 0.911), ^Table I^	
(2) Mino Belt (*Kinki & Chubu districts*)		
*Kamiaso conglomerate*	2.613)	2.55,24) 2.0,24) 1.3,24) 0.9224)
*Kiso*	2.3-1.2	
(Miso-gawa Complex20))	2.3-2.15	
(Kyogatake Complex20))	1.6	
(Shima-shima-dani Complex20))	2.0-1.6	
(Sawando Complex20))	1.35, 1.18	
(3) Ashio Belt	2.25-1.1	
*Tsukuba district*	1.8-1.65	
*southern Ashio*	1.85-1.6	
*northern Ashio*	1.6-1.4516)	
*southern Yamizo*	2.25-2.0	
*northern Yamizo*	1.36, 1.13	

*1 Osanai, Y. unpublished data,

*2 zircons in granitoids,

*3 zircons in metadiabase, Iizumi, S. unpublished data, −: Tdms can’t be calculated due to high ^143^Nd/^144^Nd and ^147^Sm/^144^Nd ratios (see Table I).

## References

[1] Ichikawa K. (1990) Introduction: Pre-Cretaceous terranes of Japan. In Pre-Cretaceous Terranes of Japan (eds. Ichikawa K..). Publ. IGCP Project 224, Nippon Insatsu Shuppan Co. Ltd., Osaka, pp. 1–11.

[2] Isozaki Y. Maruyama S. (1991). Studies on orogeny based on plate tectonics in Japan and new geotectonic sub-division of the Japanese Island. J. Geography 100, 697–761.

[3] Nishimura Y. (1990). “Sangun metamorphic rocks”; terrane problem. In Pre-Cretaceous Terranes of Japan (eds. Ichikawa K..). Publ. IGCP Project 224, Nippon Insatsu Shuppan Co. Ltd., Osaka, pp. 63–79.

[4] Isozaki Y. (1997) Contrasting two types of orogen in Permo-Triassic Japan; accretionary versus collisional. The Island Arc 6, 2–24.

[5] DePaolo D. J. (1988) Neodymium Isotope Geochemistry. Springer-Verlag, Berlin-Heidelberg, pp. 1–187.

[6] Milisenda C. C. Liew T. C. Hofmann A. W. Kröner A. (1988) Isotopic mapping of age provinces in Precambrian high-grade terrains: Sri Lanka. J. Geol. 96, 608–615.

[7] Chen Y. Yang Z. (2000) Nd model ages of sedimentary profile from the northwest Yangtze Craton, Guangyuan, Sichuan province. China and their geological implication. Geochem. J. 34, 263–270.

[8] Rollinson H. R. (1993) Using Geochemical Data. Longman Singapore Publs. Ltd., Singapore, pp. 1–352.

[9] Yamana S. (1988) The evolution of sialic crust deduced from Nd isotope: a comparison of the southern Korean Peninsula and the Inner Zone of Southwest Japan. Dr. thesis, Tokyo Univ., pp. 1–165.

[10] Morris P. A. Kagami H. (1989) Nd and Sr isotope systematics of Miocene to Holocene volcanic rocks from Southwest Japan; volcanism since the opening of the Japan Sea. Earth Planet. Sci. Lett. 92, 335–346.

[11] Kagami H. Iizumi S. Tainosho Y. Owada M. (1992) Spatial variations of Sr and Nd isotope ratios of Cretaceous-Paleogene granitoid rocks, Southwest Japan Arc. Contrib. Mineral. Petrol. 112, 165–177.

[12] Tanaka S. (1992) Origin and the early Mesozoic granitic rocks in the Hida Terrane, Japan, its implication for evolution of the continental crust. J. Sci. Hiroshima Univ. Ser. C 9, 435–493.

[13] Shimizu H. Lee S. G. Masuda A. Adachi M. (1996) Geochemistry of Nd and Ce isotopes and REE abundances in Precambrian orthogneiss clasts from the Kamiaso conglomerate, central Japan. Geochem. J. 30, 57–69.

[14] Hamamoto T. Osanai Y. Kagami H. (1999) Sm-Nd, Rb-Sr, and K-Ar geochronology of the Higo metamorphic terrane, west-central Kyushu, Japan. The Island Arc 8, 323–334.

[15] Owada M. Kamei A. Yamamoto K. Osanai Y. Kagami H. (1999) Spatial-temporal variations and origin of granitic rocks from central to northern part of Kyushu, Southwest Japan. Mem. Geol. Soc. Japan 53, 349–363.

[16] Rezanov A. I. Shuto K. Iizumi S. Shimura T. (1999) Sr and Nd isotopic and geochemical characteristics of Cretaceous-Paleogene granitoid rocks in the Niigata area, the northernmost part of the Southwest Japan. Mem. Geol. Soc. Japan 53, 269–286.

[17] Yuhara M. Kagami H. Nagao K. (2000) Geochronological characterization and petrogenesis of granitoids in the Ryoke belt, Southwest Japan Arc; constraints from K-Ar, Rb-Sr and Sm-Nd systematics. The Island Arc 9, 64–80.

[18] Kagami H. Yokose H. Honma H. (1989) ^87^Sr/^86^Sr and ^143^Nd/^144^Nd ratios of GSJ rock reference samples; JB-1a, JA-1 and JG-1a. Geochem. J. 23, 209–214.

[19] Tanaka T. Togashi S. Kamioka H. Amakawa H. Kagami H. Hamamoto T. Yuhara M. Orihashi Y. Yoneda S. Shimizu H. Kunimaru T. Takahashi K. Yanagi T. Nakano T. Fujimaki H. Shinjo R. Asahara Y. Tanimizu M. Dragusanu C. (2000) JNdi-1; a neodymium isotopic reference in consistency with LaJolla neodymium. Chem. Geol. 168, 279–281.

[20] Takeuchi M. Nakano S. Harayama S. Otsuka T. (1998) Geology of the Kiso-Fukushima district. Quadrangle Series (1: 50,000). Geol. Surv. Japan, Tsukuba.

[21] Miyamoto T. Yanagi T. (1996) U-Pb dating of detrital zircons from the Sangun metamorphic rocks, Kyushu, Southwest Japan; an evidence for 1.9–2.0 Ga granite emplacement in the provenance. Geochem. J. 30, 261–271.

[22] Tsutsumi Y. Yokoyama K. Terada K. Sano Y. (2000) SHRIMP U-Pb dating of zircons in the sedimentary rocks from the Akiyoshi and Suo Zones, Southwest Japan. J. Petr. Sci. 95, 216–227.

[23] Yamashita K. Yanagi T. (1994) U-Pb and Rb-Sr dating of the Oki metamorphic rocks, the Oki Island, Southwest Japan. Geochem. J. 28, 333–339.

[24] Sano Y. Hidaka H. Terada K. Shimizu H. Suzuki M. (2000) Ion microprobe U-Pb zircon geochronology of the Hida gneiss: finding of the oldest minerals in Japan. Geochem. J. 34, 135–154.

[25] Herzig C. T. Kimbrough D. L. Tainosho Y. Kagami H. Iizumi S. Hayasaka Y. (1998) Late Cretaceous U/Pb zircon ages and Precambrian crustal inheritance in Ryoke granitoids, Kinki and Yanai districts, Japan. Geochem. J. 32, 21–32.

[26] Ishizaka K. (1969) U-Th-Pb ages of zircon from the Ryoke metamorphic terrain, Kinki district. J. Jap. Assoc. Min. Petr. Eco. Geol. 62, 191–197.

[27] Tanaka T. Hoshino M. (1987) Sm-Nd ages of the Oki metamorphic rocks and their geological significance. Abst. 94th Annu. Meet. Geol. Soc. Japan (Osaka), p. 492.

[28] Ishiwatari A. (1985) Granulite-facies metacummulates of the Yakuno ophiolite; evidence for unusually thick oceanic crust. J. Petrol. 26, 1–30.

[29] Shibata K. Adachi M. (1974) Rb-Sr whole-rock ages of Precambrian metamorphic rocks in the Kamiaso conglomerate from central Japan. Earth Planet. Sci. Lett. 21, 277–287.

[30] Lee S.-G. Masuda A. Shimizu H. Song Y. S. (2001) Crustal evolution history of Korean Peninsula in East Asia; the significance of Nd, Ce isotopic and REE data from the Korean Precambrian gneisses. Geochem. J. 35, 175–187.

[31] Inomata M. (1999) Ages of the basement rocks of the northern part of the Korea Peninsula. Abst. 106th Annu. Meet. Geol. Soc. Japan (Nagoya), p. 114.

[32] Kagami H. Yuhara M. Iizumi S. Tainosho Y. Owada M. Ikeda Y. Okano O. Ochi S. Hayama Y. Nureki T. (2000) Continental basalts in the accretionary complexes of the Southwest Japan Arc; constraints from geochemical and Sr and Nd isotopic data of metadiabase. The Island Arc 9, 3–20.

[33] Roser B. P. Korsch R. J. (1986) Determination of tectonic setting of sandstone-mudstone suites using SiO_2_ content and K_2_O/Na_2_O ratio. J. Geol. 94, 635–650.

[34] Kim O. J. (1987) Tectonic evolution. In Geology of Korea (ed. Lee D. S.). Kyohak-sa Publs. Co., Seoul, pp. 252–263.

[35] Inomata M. (1998) Archean rocks of the Korean Peninsula. Abst. IGCP Project 221 Symposium, Wuhan, China, pp. 40–42.

[36] Suzuki K. Adachi M. Kato T. (1999) CHIME ages of basement rocks in the southern part of the Korean Peninsula. Abst. 106th Annu. Meet. Geol. Soc. Japan (Nagoya), p. 7.

[37] Kim Y. U. Inomata M. Sato S. Aoki H. (1995) Tectonic importance of North Korea in East Asia; Archean Era. J. School Marine Sci. Tech. Tokai Univ. 40, 73–80.

[38] Osanai Y. Hamamoto T. Kamei A. Owada M. Kagami H. (1996) High-temperature metamorphism and crustal evolution of the Higo metamorphic terrane, central Kyushu, Japan. In Tectonics and Metamorphism (eds. Shimamoto T..), Soubun Co. Ltd., Tokyo, 113–124.

[39] Suzuki K. Adachi M. Takagi H. Osanai Y. (1998) CHIME monazite age of the Higo metamorphic rocks. Abst. 105th Annu. Meet. Geol. Soc. Japan (Matsumoto), p. 214.

